# Analogies between optical propagation and heat diffusion: applications to microcavities, gratings and cloaks

**DOI:** 10.1098/rspa.2015.0143

**Published:** 2015-11-08

**Authors:** C. Amra, D. Petiteau, M. Zerrad, S. Guenneau, G. Soriano, B. Gralak, M. Bellieud, D. Veynante, N. Rolland

**Affiliations:** 1 Aix Marseille Université, Institut Fresnel, CNRS, Ecole Centrale Marseille, Faculté des Sciences et Techniques de St Jérôme, 13397 Marseille Cedex 20, France; 2 Université Montpellier 2, CNRS, LMGC, Montpellier, France; 3 Ecole Centrale Paris, CNRS, EM2C, Paris, France; 4 Université de Lille 1, CNRS, IEMN, Lille, France

**Keywords:** optical propagation, heat diffusion, admittance formalism, microcavities, diffraction gratings, cloaks

## Abstract

A new analogy between optical propagation and heat diffusion in heterogeneous anisotropic media has been proposed recently by three of the present authors. A detailed derivation of this unconventional correspondence is presented and developed. In time harmonic regime, all thermal parameters are related to optical ones in artificial metallic media, thus making possible to use numerical codes developed for optics. Then, the optical admittance formalism is extended to heat conduction in multilayered structures. The concepts of planar microcavities, diffraction gratings and planar transformation optics for heat conduction are addressed. Results and limitations of the analogy are emphasized.

## 1. Introduction

Optics and heat conduction have often been associated, because optical devices and systems generally have to face thermal effects. When the electromagnetic field interacts with dense matter, optical losses are converted into heat, which generates conductive, convective and radiative transfer. These well-known photo-induced thermal effects have motivated a number of papers, for instance in the field of photothermal microscopy, laser-induced damage and temperature insensitive devices [1–6]. Specific models have then been developed to take into account heat transfer inside multilayers [1–6]. Moreover, thermal emission has been deeply investigated, within the framework of nanoscale photonic structures [7–15].

A novel association of optics and heat has been proposed recently [16–28]. It is based on an analogy between the governing equations of propagation for optics and diffusion for heat. The correspondence is not intuitive according to the markedly different nature of optical propagation and thermal diffusion phenomena. Nevertheless, an analogy can be unveiled in time harmonic regime when metallic media are considered in optics. Under these conditions, electromagnetic fields decay exponentially fast away from a metal–dielectric interface, so that their behaviour is somewhat reminiscent of a temperature field undergoing a fast decay away from a heat source. Such an analogy has been first used in the design of thermal cloaks, heat concentrators and rotators [16–28], using geometric transformation methods previously applied to electromagnetics [29,30]. A series of papers subsequently emerged and confirmed tools of transformation optics can be applied to control other diffusion processes [31,32].

In this work, we further explore the unconventional analogy between transformation optics and thermodynamics which has been first proposed in reference [16]. Correspondences are emphasized between optical and thermal fields, i.e. (vector) electric fields and (scalar) temperature, (vector) magnetic fields and (vector) heat flux. All thermal parameters are related to optical parameters in artificial metallic media, thus making it possible to reintroduce the optical concepts of effective index and complex admittance for heat conduction. More generally, all numerical codes developed for optics (multilayers, gratings, microcavities, scattering and diffraction, transformation optics …) can be used. The plan of this article is as follows: the whole optical admittance formalism [33–38] is first extended to thermal conduction, and deep analogies are drawn with multilayered electromagnetic structures in order to predict thermal properties in multilayers. Next, the concepts of planar microcavities, diffraction gratings and planar cloaks are addressed in the frame of heat conduction. A particular attention is paid to the mathematical correspondences and limitations between metal optics and heat conduction, including fields and energy balances, together with the extended optical admittance formalism for conduction.

## 2. Optical propagation versus heat conduction

### (a) Spatio-temporal regime

Free space optical propagation in linear, isotropic and homogeneous materials can be classically modelled using the governing equation [38–43]:

2.1 $$\Delta \text{E}-\varepsilon \mu^{*}{\;}_{t}\partial^{2}\text{E}/\partial t^{2}={\text{S}}_{\mathrm{opt}}(\text{J},q)$$

with **E** the vector electric field, *ε* and *μ* the temporal permittivity and permeability, **J** and *q* the density of currents and charges and **S**_*opt*_ a source term resulting from these densities. In equation (2.1), the convolution product (*_t_) involves time (*t*) and takes into account the material inertia, that is, the time delay between the excitation of material and its optical response. Note the second-order derivation versus time of the electric field (elliptic equation).

On the other hand, heat conduction [43–46] involves a first-order derivation versus time (parabolic equation). We will here consider that conduction satisfies the following equation in linear, isotropic and homogeneous media as

2.2 $$\Delta T-\frac{1}{a}\frac{\partial T}{\partial t}=-\frac{S}{b}=S_{\mathrm{th}}$$

with *T* the temperature and *a*,*b* the thermal parameters, that is, diffusivity and conductivity, respectively. The source term *S* is the bulk density of heat power. Note that in equation (2.2) the temperature dependence of the thermal parameters is neglected.

In addition to the discrepancy in time derivation order, other straightforward differences can be emphasized between optical propagation and heat diffusion, which are

— heat equation is scalar, whereas optics equation is vectorial; however, one can consider optics equations on each basis vector, and
— optical parameters (*ε*,*μ*) vary with time and hence will exhibit a frequency dispersion, whereas this is not the case for the thermal parameters in equation (2.2).

Another difference lies in the fact that the temperature (or a flux condition) is most often fixed at the domain frontier, a supplementary condition not present in free space optics. However, in the absence of sources, heat flux and temperature are continuous at interfaces, as it is for the tangential electromagnetic fields.

In equations (2.1) and (2.2), all fields vary with time (*t*) and space location *ρ*=(**r**,*z*)=(*x*,*y*,*z*). It is interesting to recall the Green's functions *G* of equations (2.1) and (2.2) that give the particular solutions in the form $G_{t}^{*}S_{\mathrm{opt}}\;\text{or}\;G_{t}^{*}S_{\mathrm{th}},$ with

2.3 $$G_{\mathrm{opt}}(\rho ,t)=\left(-\frac{1}{4\pi \rho }\right)\delta (t-\rho \sqrt{\left(\varepsilon \mu \right)})\;\text{in optics}$$

and

2.4 $$\;G_{\mathrm{th}}=\left(\frac{1}{8b\surd a}\right)\left[\frac{1}{{\left(\pi t\right)}^{3/2}}\right]\;\text{exp}\left[\frac{-\rho^{2}}{4\text{at}}\right]H(t)\;\text{in conduction,}$$

with *ρ*=|**ρ**|, *H* the Heaviside function and *δ* the Dirac distribution. Equation (2.3) is given under the assumption that the optical material is not dispersive, and shows that the optical wavefront propagates at velocity *v*=1/√(*εμ*) and is located at time *t* at the sphere surface of radius *ρ*=*vt* where the whole energy is located. On the other hand, equation (2.4) is for a diffusion process, so that the energy is spread within the whole heated volume of radius *ρ*=2√(at) which increases with time. Note that (2.4) does not involve any Dirac distribution: strictly speaking, heat would have already diffused everywhere at arbitrary time (*t*≠0⇒*G*_*th*_≠0 ∀*ρ*), owing to the Gaussian nature of *G*_*th*_.

In what follows, we now consider the propagation or conduction equations in isotropic homogeneous regions free of sources, what we write as the homogeneous equations

2.5 $$\Delta \text{E}-\varepsilon \mu {{\;}^{*}}_{t}\partial^{2}\text{E}/\partial t^{2}=0\;\text{for optics, on each basis vector}\;(\text{E}={\text{E}}_{x},{\text{E}}_{y}\;\text{or}\;{\text{E}}_{z})$$

and

2.6 $$\Delta T-\frac{1}{a}\frac{\partial T}{\partial t}=0\;\text{for conduction.}$$

Moreover, we will be interested in a temperature elevation resulting from a transient heat source, similar to what happens when the heat source results from the absorption of a modulated laser beam. Following the superposition properties of linear systems, the total temperature satisfies *T*=*T*_*a*_+*T*^′^ with *T*_*a*_ owing to the environment, and *T*^′^ resulting from the heat source given as *S*=*S*_0_[1+*h*(*t*)] with |*h*(*t*)|<1. This last temperature *T*^′^ can be written as *T*^′^=*T*_1_+*T*_2_, where *T*_1_ is a steady-state temperature and results from *S*_0_, whereas *T*_2_ is a transient temperature and results from *S*_0_*h*(*t*). In this work, we are actually interested in this last transient temperature *T*_2_.

### (b) Harmonic regime

For the sake of simplicity, we use Fourier transform rather than Laplace transform, despite the differences in the two transforms. Another reason is that a modulated laser beam will create optical absorption (and so heat power) resulting in a periodic *h*(*t*) function which can be written as a Fourier series. Hence, we now consider Fourier transform versus time of the optical and heat equations, with *ω* the temporal pulsation, conjugate of the time variable, and *f*=*ω*/2*π* the temporal frequency. The result is well known and written as

2.7 $$\Delta \overset{ˇ}{u}+k^{2}(\omega )\overset{ˇ}{u}=0,$$

with *k* the wavenumber (or conduction number by analogy with optics), *u* the scalar electric field or temperature and *u* its Fourier transform defined as

2.8a $$\overset{ˇ}{u}(\rho ,f)=\int_{t}u(\rho ,t)\;\;\text{exp}(j2\pi ft)\text{d}t$$

and

2.8b $$u(\rho ,t)=\int_{f}\overset{ˇ}{u}(\rho ,f)\;\text{exp}(-j2\pi ft)\text{d}f$$

Equation (2.7) shows that both fields (*u*=*E* or *T*) satisfy a similar harmonic equation in the Fourier space, taking into account the specific dispersion law of the *k* function, that is

2.9 $$k^{2}(\omega )=\omega^{2}\overset{ˇ}{\varepsilon }(\omega )\overset{ˇ}{\mu }(\omega )\Leftrightarrow k=\omega \sqrt{\left[\overset{ˇ}{\varepsilon }(\omega )\overset{ˇ}{\mu }(\omega )\right]}\;\text{in optics}$$

and

2.10 $$k^{2}(\omega )=\frac{j\omega }{a}\Leftrightarrow k=(1+j)\surd [\omega /2a]\;\text{in conduction.}$$

In other words, the dispersion law *k*(*ω*) is the memory of the *p*th order of time derivation (*p*=1 for conduction, *p*=2 for optics). Note in (2.9) that $\overset{ˇ}{\varepsilon }(\omega )$ and $\overset{ˇ}{\mu }(\omega )$ are the Fourier transforms of the optical functions (*ε*,*μ*), whereas in (2.10), the diffusivity parameter *a* is not dispersive under the thermal approximation. Hence, the *k* dispersion behaves as $\sqrt{\omega }$ for conduction, whereas in optics, it depends on the permeability and permittivity frequency dispersion law; however, as a first approximation, the *k* dispersion in optics can be considered to follow *ω* in a narrow bandwidth.

Above all, a major point to be emphasized in the harmonic regime concerns the *k* value at a given frequency. Indeed, in optics, such a value can be real or complex, depending upon whether dielectrics or metals are under study. On the other hand, the *k* value is necessarily complex in conduction for reasons following (2.7)–(2.10), so that heat diffusion can be considered to be analogous to optical propagation (more exactly, electric field attenuation) in artificial metallic media. Furthermore, following (2.10), the equivalent metal should be specific with identical real and imaginary parts in the refractive index *n* (defined as *k*=*k*_0_ *n* in optics).

## 3. Optical admittance formalism for multilayers

### (a) Multilayer optics: basics

**Figure 1.**
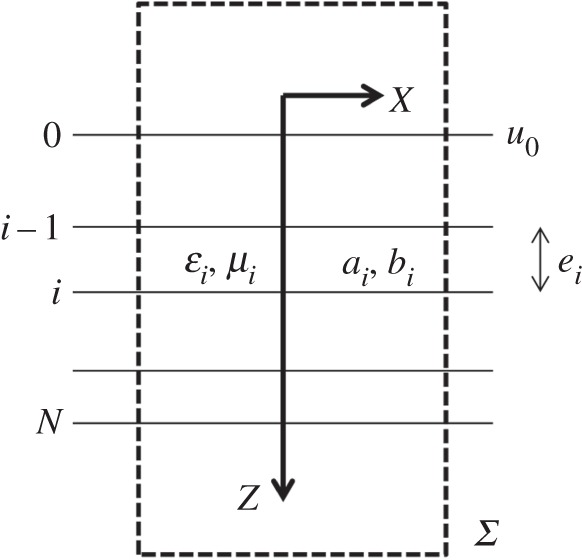
Geometry of the multilayer for optics or heat conduction.

In multilayer optics (figure 1), the admittance formalism is commonly used to predict optical properties and to design antireflective coatings, beam splitters, polarizing devices, mirrors and narrow-band filters [33–38]. It is based on the complex admittance, a key function that often simplifies the calculation and allows for analytical design. This admittance function *Y* is given for a particular polarization (transverse electric *S* or transverse magnetic *P*) as

3.1 $$\hat{\text{H}}=Yz^{\wedge }\hat{\text{E}},$$

with $\hat{\text{H}}$ and $\hat{\text{E}}$ the tangential components of the electric and magnetic polarized field, and **z** the direction normal to the stack. Note here that all fields are harmonic (monochromatic) and result from a single illumination incidence at the top surface of the stack; in other words, the admittance is defined after double Fourier transform (versus time *t* and versus transverse coordinate **r**) of the fields; we will come back to this point in the next section, where we re-derive the admittance formalism.

In the case of a stationary wave within the stack, the admittance varies with the *z* altitude; on the other hand, when the wave is only progressive or retrograde, what may happen in free space in the surrounding media, it is constant and reduced to the effective index ±*m*, that is

3.2a $${\hat{\text{H}}}^{+}=m{\text{z}}^{\wedge }{\hat{\text{E}}}^{+}\;\text{for}\;\text{a}\;\text{progressive}\;\text{wave}$$

and

3.2b $${\hat{\text{H}}}^{-}=-m{\text{z}}^{\wedge }{\hat{\text{E}}}^{-}\;\text{for}\;\text{a}\;\text{retrograde}\;\text{wave.}$$

Equations (3.1) and (3.2) are given for each polarization of the optical field, which means that both effective indices and admittances are polarization (and incidence, wavelength) dependent. Moreover, we have assumed to be in the far field, so that the electric field **E** is driven by two transverse polarization modes.

The effective indices are known from the illumination conditions, and enable to initiate a sequence for the admittance extraction throughout the whole stack (see next section). They are given as

3.3a $$m=\left(\frac{1}{\eta_{0}\mu_{r}}\right)\frac{n\alpha }{k}\;\text{for transverse electric (TE) polarization}$$

and

3.3b $$m=\left(\frac{1}{\eta_{0}\mu_{r}}\right)\frac{nk}{\alpha }\;\text{for transverse magnetic (TM) polarization,}$$

with *η*_0_=√(*μ*_0_/*ε*_0_) the vacuum impedance, $n=\surd ({\overset{ˇ}{\varepsilon }}_{r}{\overset{ˇ}{\mu }}_{r})$ the refractive index, $\left({\overset{ˇ}{\varepsilon }}_{r},{\overset{ˇ}{\mu }}_{r}\right)$ the complex relative permittivity and permeability, *α*=√(*k*^2^−*σ*^2^) and *σ* the spatial pulsation which can be connected in transparent media to a propagation direction *θ* (illumination angle), that is

3.4 σ=k sinθ=2πν⇒α=k cosθ

with *ν* the spatial frequency; in the case of plane waves (*σ*<*k*), relation (3.3) is turned into: $m\;=\;(1/\eta_{0}\mu_{r})\;n\cos\theta$ for TE polarization and $m=(1/\eta_{0}\mu_{r})\;n/\cos\theta$ for TM polarization.

All relations (3.1)–(3.4) are valid for plane, evanescent or dissociated waves.

### (b) Conduction in multilayers

Let us now derive the admittance formalism in a few lines, and show how it can also be applied to conduction. For the sake of simplicity (i.e. to avoid the double Fourier transform addressed in the next sections), we start with a problem invariant with respect to the transverse coordinates (*x*,*y*), so that relation (2.7) only involves *z* and *ω* variables

3.5 $$\frac{\partial^{2}\overset{ˇ}{u}}{\partial z^{2}}+k^{2}(\omega )\overset{ˇ}{u}=0.$$

In optics, the unique *z*-dependence corresponds to the case of a plane wave illuminating the stack at normal incidence (*σ*=0). The solution of (3.5) is given in each layer (*i*=1,*N*) by

3.6 $${\overset{ˇ}{u}}_{i}(z,\omega )={\overset{ˇ}{u}}_{i}^{+}(\omega )\text{ exp[}jk_{i}(\omega )z\text{] }+{\overset{ˇ}{u}}_{i}^{-}(\omega )\text{ exp[}-jk_{i}(\omega )z\text{],}$$

where the field is *E* or *T* in the layer. The next step in optics to complete the admittance formalism is based on the fact that the magnetic field $\overset{ˇ}{\text{v}}$ follows the same analytical expression (3.6), and that the electric and magnetic components are linked through (3.1) and (3.2), that is

3.7 $$\begin{array}{rl} {\overset{ˇ}{\text{v}}}_{i}(z,\omega ) & ={\overset{ˇ}{\text{v}}{\;}^{+}}_{i}(\omega )\;\text{exp}[jk_{i}(\omega )z]+{\overset{ˇ}{\text{v}}}_{i}^{-}(\omega )\text{ exp[}-jk_{i}(\omega )z\text{]} \end{array}$$

3.8 $$\begin{array}{rl} {\overset{ˇ}{\text{v}}}_{i}^{+} & =m_{i}{\text{z}}^{\wedge }{\overset{ˇ}{\text{u}}}_{i}^{+}\;\text{and}\;{\overset{ˇ}{\text{v}}}_{i}^{-}=-m_{i}{\text{z}}^{\wedge }{\overset{ˇ}{\text{u}}}_{i}^{-} \end{array}$$

3.9 $$\begin{array}{rl} \text{and}\;{\overset{ˇ}{\text{v}}}_{i} & ={\overset{ˇ}{\text{v}}}_{i}^{+}+{\overset{ˇ}{\text{v}}}_{i}^{-}=Y_{i}{\text{z}}^{\wedge }{\overset{ˇ}{u}}_{i} \end{array}$$

The last step consists of combining (3.8)–(3.9) and writing the continuity of the tangential electric and magnetic fields in the absence of sources, which results in a matrix formalism to pass from one interface to the next as

3.10 $$\left({\text{z}}^{\wedge }{\overset{ˇ}{\text{u}}}_{i-1},{\overset{ˇ}{\text{v}}}_{i-1})=M_{i}({\text{z}}^{\wedge }{\overset{ˇ}{\text{u}}}_{i},{\overset{ˇ}{\text{v}}}_{i})\;\text{with}\;\text{the}\;\text{matrix}\;M_{i}=(s_{\mathrm{kl}}^{i}\right)$$

and

3.11 $$s_{11}^{i}=s_{22}^{i}=\text{cos}\delta_{i},s_{12}^{i}=-j\frac{\text{sin}\delta_{i}}{m_{i}},s_{21}^{i}=-jm_{i}\;\text{sin}\delta_{i}\;\text{and}\;\delta_{i}=k_{i}e_{i},$$

with *e*_*i*_ the thickness of layer *i*.

Now, we can check that the similarity with the conduction process is immediate. First of all, we need two quantities that are continuous throughout the whole stack; in the absence of singular sources, these quantities are the harmonic temperature $\overset{ˇ}{T}$ and the scalar (*z*-projection) heat flux density $\overset{ˇ}{h}$ given by

3.12 $$\overset{ˇ}{h}=-b\frac{\partial \overset{ˇ}{T}}{\partial z}\;\text{with}\;\frac{\partial^{2}\overset{ˇ}{h}}{\partial z^{2}}+k^{2}(\omega )\overset{ˇ}{h}=0.$$

Hence, the temperature plays the role of the electric field in (3.6), whereas the heat flux replaces the magnetic field in (3.7). However, this last condition must be further clarified to determine which quantity replaces the effective index m in (3.8). By analogy with optics (relation (3.2)a,b)), this last quantity is defined for conduction as

3.13 $${\overset{ˇ}{h}}^{+}=m{\overset{ˇ}{T}}^{+}\;\text{and}\;{\overset{ˇ}{h}}^{-}=-m{\overset{ˇ}{T}}^{-}$$

The result is immediate from (3.7), that is

3.14 m=−jkb,

and the admittance follows within the stack as

3.15 $$\overset{ˇ}{h}(\omega ,z)={\overset{ˇ}{h}}^{+}+{\overset{ˇ}{h}}^{-}=Y(\omega ,z)\overset{ˇ}{T}(\omega ,z).$$

At this stage, the correspondence is complete and the matrix formalism (3.10) and (3.11) can be used either in optics or conduction in the harmonic regime. Note that two slight modifications were necessary to reach this one-to-one correspondence, which are

— the optical parameter $k_{i}=\omega \surd [{\overset{ˇ}{\varepsilon }}_{i}(\omega ){\overset{ˇ}{\mu }}_{i}(\omega )]$ is replaced by the conduction parameter *k*_*i*_=(1+*j*) √[*ω*/2*a*_*i*_], and
— the optical effective index *m*_*i*_=*n*_*i*_/(*η*_0_*μ*_*ri*_) is replaced by that of conduction *m*_*i*_=−*jk*_*i*_*b*_*i*_.

### (c) Wavelength, diffusion length and phase velocity

From equations (2.8) and (3.6), the field (T or E) can be reconstructed in the layers as the sum of elementary components. In the substrate, these components take the form

3.16 $${\overset{ˇ}{u}}_{\mathrm{el}}=\text{real}\{{\overset{ˇ}{u}}^{+}(\omega )\text{exp[}j(k(\omega )z-\omega t)\text{]}\}=\;\text{exp}\left(-k^{\prime \prime }z\right)|{\overset{ˇ}{u}}^{+}|\text{cos}[k^{\prime }z-\omega t+\psi^{+}]$$

with ${\overset{ˇ}{u}}^{+}=|{\overset{ˇ}{u}}^{+}|\exp(j\psi^{+})$ and *k*=*k*^′^+*j* *k*^′′^. The real exponential is for the field envelope, whereas the cosine term allows one to retrieve a spatial period *λ*=2*π*/*k*^′^ (the wavelength in optics) and a phase velocity *v*=*ω*/*k*^′^ (light velocity in optics). Equation (3.16) shows that in conduction the (pseudo)spatial period *λ*_*th*_ is proportional to the diffusion length

3.17 L=√(2a/ω),

defined at 1/*e* for the temperature amplitude, that is *λ*_*th*_=2*π* *L*. Also, quantity

3.18 $$v_{\mathrm{th}}=\surd (2a\omega ),$$

is analogous to the phase velocity of optics. However, we do not extend the analogy to the refractive index, because this index involves the vacuum property in optics (where propagation occurs), whereas conduction fails in vacuum (another reference material should be used).

### (d) Two-dimensional formalism for spatial wave packet

Now, we take into account the spatial distribution of the field, which means that we drop the unique *z*-dependence to recover a **ρ**=(**r**,*z*) variable, with **r** = (*x*,*y*). Such an extension to a three-dimensional geometry is immediate if we consider, in addition to the frequency wave packet (Fourier transform versus time), a spatial wave packet (Fourier transform versus space coordinate **r**). This second Fourier transform is given as

3.19a $$\hat{u}(\nu ,z,\omega )=\int_{\text{r}}\overset{ˇ}{u}(\text{r},z,\omega )\text{ exp}(-j2\pi \nu .\text{r})\text{ d}\text{r}$$

and

3.19b $$\overset{ˇ}{u}(\text{r},z,\omega )=\int_{\nu }\hat{u}(\nu ,z,\omega )\;\text{exp}(j2\pi \nu .\text{r})\text{d}\nu$$

with ***ν*** the spatial frequency, the Fourier variable conjugated of **r**. Such double Fourier transform $\hat{u}$ is governed by the same equation (3.5) as the single Fourier transform $\overset{ˇ}{u}$, except that *k*(*ω*) must be replaced by *α*(*ω*)=√(*k*^2^−*σ*^2^), with ***σ***=2*π****ν*** the spatial pulsation. We obtain

3.20 $$\frac{\partial^{2}\hat{u}}{\partial z^{2}}+\alpha^{2}(\sigma ,\omega )\hat{u}=0.$$

In each layer, the field in this second Fourier plane takes the form

3.21 $${\hat{u}}_{i}(\nu ,z,\omega )={\hat{u}}_{i}^{+}(\nu ,\omega )\text{exp}[j\alpha_{i}(\nu ,\omega )z]+{\hat{u}}_{i}^{-}(\nu ,\omega )\;\text{exp}[-j\alpha_{i}(\nu ,\omega )z],$$

so that the elementary components that enable to rebuild the original (spatio-temporal) field can once again be written in the substrate as

3.22 $${\hat{u}}_{\mathrm{el}}=\text{ real}\{{\hat{u}}^{+}(\sigma ,\omega )\text{exp}\{j[\sigma .\text{r}+\alpha (\sigma ,\omega )z-\omega t]\}=\;\text{exp}(-\alpha^{\prime \prime }z)|{\hat{u}}^{+}|\text{cos}[\sigma .\text{r}+\alpha^{\prime }z-\omega t+\phi^{+}]$$

with ${\hat{u}}^{+}=|{\hat{u}}^{+}|\;\text{exp}\;(j\phi^{+})$ and *α*=*α*^′^+*jα*^′′^. In other words, this works like optics at oblique incidence and the *k* parameter only has to be replaced by *α*=√(*k*^2^−***σ***^2^). The salient consequence for the matrix formalism (3.10) and (3.11) following the previous section is that the effective index for heat conduction (relation (3.14) must also be modified as

3.23 m=−jαb.

Regarding the spatial and temporal period, *k*^′^ must be replaced by real(*α*). At last, the original (real) field is reconstructed with a double inverse Fourier transform over temporal (*f*) and spatial (***ν***) frequency.

## 4. Energy balance

To be complete, our correspondences should also involve an energy balance for both optical propagation and heat conduction. This condition is written below within a domain *Ω* limited by a closed surface *Σ* with unit outward normal **n**(figure 1).

### (a) Temporal regime

In optics, we obtain from Maxwell's equations in the spatio-temporal regime

4.1 $$-\int_{\Omega }\text{J}.\text{E}\text{d}v=\int_{\Sigma }\text{P}.\text{n}\;\text{d}\;\Sigma +\frac{\partial }{\partial t}\left(\int_{\Omega }w\text{d}\Omega \right)\Leftrightarrow F=\Phi +A,$$

with **P** = **E**^∧^**H** the real Poynting vector, *F* the optical power provided by sources within *Ω*,*Φ* the Poynting flux through the surface *Σ*, and *A* the variation of internal energy (or absorption) within *Ω*, with d*A*/d*Ω*=∂*w*/∂*t*=**H**.∂/∂*t*(μ*H)+**E**.∂/∂*t*(ε*E). As usual, the density of electromagnetic energy reduces to *w*=(1/2)[**E**^2^+**H**^2^] when the material inertia is neglected.

For purely heat conduction, the balance is directly obtained from the governing equation integrated over *Ω*

4.2a $$S=\text{div}(\text{h})+\left(\frac{b}{a}\right)\frac{\partial T}{\partial t}\;\text{with}\;\text{h}=-b\text{grad}T$$

and

4.2b $$\int_{\Omega }S\text{ d}\Omega =-\int_{\Sigma }\text{b}\;\text{grad}\text{T}.\text{n}\;\text{d}\;\Sigma +\frac{\partial }{\partial t}\left[\int_{\Omega }\left(\frac{b}{a}\right)T\;\text{d}\Omega \right]\Leftrightarrow F=\Phi +A.$$

with *F* the heat power provided by the source, *Φ* the heat flux through *Σ* and *A* the variation of internal energy, with: (*b*/*a*) ∂ T /∂*t*=∂ w/∂*t*= d *A*/d*Ω*. In other words, the density of electromagnetic energy is here replaced by a quantity proportional to the temperature, with *b*/*a*=*γ*C the product of mass bulk density (*γ*) and heat capacity (C).

### (b) Fourier analysis

Such balances (4.1) and (4.2) must now be rewritten in the Fourier planes. In optics, quadratic (intensity) detectors are known to realize a time average process of the square fields, owing to their low temporal response with respect to optical periods. Such specificity allows one to transform the spatio-temporal balance (4.1) into a single harmonic balance at each temporal pulsation *ω* (first Fourier plane), that is

4.3 $$-\int_{\Omega }\overset{ˇ}{\text{J}}\cdot {\overset{ˇ}{\text{E}}}^{*}\text{d}\Omega =2\int_{\Sigma }\Pi .\text{n}\;\text{d}\;\Sigma -j\omega \left\{\int_{\Omega }\left[\overset{ˇ}{\varepsilon }{\left|\overset{ˇ}{\text{E}}\right|}^{2}+\overset{ˇ}{\mu }{\left|\overset{ˇ}{\text{H}}\right|}^{2}\right]\text{d}\Omega \right\}$$

with ***Π*** the complex harmonic Poynting vector:

4.4 $$\Pi =\left(\frac{1}{2}\right)\overset{ˇ}{\text{E}}{{\;}^{*}}^{\wedge }\overset{ˇ}{\text{H}}.$$

Then, to reach a balance at a single spatial frequency ***ν*** in the second Fourier plane, one may use Parseval's theorem and other specific properties to find

4.5 $$-\int_{\nu ,z}\hat{\text{J}}\cdot {\hat{\text{E}}}^{*}\;\text{d}\nu \text{d}z=2\int_{\nu }\Pi^{\prime }\cdot \text{n}\;\text{d}\nu -j\omega \left\{\int_{\nu ,z}\left[\overset{ˇ}{\varepsilon }{\left|\hat{\text{E}}\right|}^{2}+\overset{ˇ}{\mu }{\left|\text{H}\right|}^{2}\right]\;\text{d}\nu \text{d}z\right\}$$

and

4.6 $$\Pi^{\prime }=\left(\frac{1}{2}\right){\hat{\text{E}}}^{*}{\;}^{\wedge }\hat{\text{H}}.$$

In a last step, considering the hermiticity of the fields, we recover the harmonic balance per unit of spatial frequency d*ν*_*x*_ d*ν*_*y*_ with respect to a surface *Σ* surrounding the whole planar multilayer with sides at infinity (figure 1)

4.7 $$-\text{real}\left\{\int_{z}\hat{\text{J}}\cdot {\hat{\text{E}}}^{*}\;\text{d}z\right\}=\text{real}\{{\left[Y|\hat{\text{E}}{|}^{2}\right]}_{z}\}+\omega \left\{\int_{z}\left[\varepsilon^{\prime \prime }{\left|\hat{\text{E}}\right|}^{2}+\mu^{\prime \prime }{\left|\hat{\text{H}}\right|}^{2}\right]\;\text{d}z\right\},$$

with εˇ″=Im(εˇ), μˇ″=Im (μˇ) and the notation [*u*]_*z*_=*u*(*z*_2_)−*u*(*z*_1_), where *z*_*i*_ are the upper and lower altitudes of *Σ* and *z*_2_>*z*_1_. Note here that we used equations (3.1) and (4.6) to reach the property: $2\Pi^{\prime }\cdot \text{n}=Y{\left|\hat{\text{E}}\right|}^{2},$ a flux quantity which is continuous throughout the stack in the absence of sources.

Addressing the harmonic balance in conduction is *a priori* simpler, because temporal frequencies are much lower than in optics, thus disregarding temporal integration by the detector is legitimate. Moreover, here the thermal parameters are assumed to be non-dispersive, which simplifies the calculation. One can directly use the Fourier transform versus time of the governing equation (4.2) to reach a complex harmonic balance at temporal pulsation *ω*

4.8 $$\int_{\Omega }\overset{ˇ}{S}\;\text{d}\Omega =-\int_{\sum }\text{b}\frac{\partial \overset{ˇ}{T}}{\partial n}\;\text{d}\Sigma -j\omega \int_{\Omega }\left(\frac{b}{a}\right)\;\overset{ˇ}{T}\text{d}\Omega .$$

Per unit of surface area d*x*d*y* parallel to the interfaces of the stack, we obtain

4.9 $$\int_{z}\overset{ˇ}{S}\text{d}z=-{\left[b\frac{\partial \overset{ˇ}{T}}{\partial z}\right]}_{z}-j\omega \int_{z}\left(\frac{b}{a}\right)\overset{ˇ}{T}\text{d}z.$$

Owing to the Hermitian property of $\overset{ˇ}{T}$ such a balance can also be limited to positive frequencies *ω* as follows

4.10 $$\text{real}\left\{\int_{z}\overset{ˇ}{S}\text{d}z\right\}=-\text{real}\left\{{\left[b\frac{\partial \overset{ˇ}{T}}{\partial z}\right]}_{z}\right\}-\text{real}\left\{j\omega \int_{z}\left(\frac{b}{a}\right)\overset{ˇ}{T}\text{d}z\right\}.$$

Now, to write the conduction balance at a single spatial frequency (second Fourier plane), *a* *z* integration after a double Fourier transform of (4.2) directly leads to

4.11 $$\int_{z}\hat{S}\;\text{d}z={\left[Y\hat{T}\right]}_{z}-\int_{z}b\alpha^{2}\hat{T}\;\text{d}z,$$

with *α*^2^=*k*^2^−***σ***^2^. Note again in (4.11) that $Y\;\hat{T}$ is a flux quantity which is continuous within the stack.

## 5. Optical admittance calculation for heat conduction

This section is devoted to numerical calculation. For that, we use an optical thin film admittance software where the *k* dispersion and the effective index were rewritten following (2.10) and (3.23). As usual [33–37], the effective index value in the substrate is the starting value of the admittance sequence within the stack, given at each interface by

5.1 $$Y_{i-1}=\frac{Y_{i}\text{cos}\delta_{i}-jm_{i}\text{sin}\delta_{i}}{\text{cos}\delta_{i}-jY_{i}\text{sin}\delta_{i}/m_{i}}.$$

A similar sequence is given for the field at interfaces

5.2 $${\hat{u}}_{i-1}={\hat{u}}_{i}\left(\text{cos}\delta_{i}-j\left(\frac{Y_{i}\text{sin}\delta_{i}}{m_{i}}\right)\right).$$

A complete *z*-variation of admittance and field can also be obtained in the layers if we replace *δ*_*i*_=*α*_*i*_*e*_*i*_ by *δ*_*i*_=*α*_*i*_*z*, with 0<*z*<*e*_*i*_. Finally, reflection (*r*) and transmission (*t*) factors can be defined in a way similar to optics, that is

5.3 $$\begin{array}{rl} r & =\frac{m_{0}-Y_{0}}{m_{0}+Y_{0}}\;\text{at the top interface,} \end{array}$$

5.4 $$\begin{array}{rl} t & =\frac{1+r}{\left[\prod_{i=1,N}(\text{cos}\delta_{i}-jY_{i}\text{sin}\delta_{i}/m_{i})\right]}\;\text{at the bottom interface} \end{array}$$

5.5 $$\begin{array}{rl} \text{and}\;r & =\frac{{\hat{u}}_{0}^{-}}{{\hat{u}}_{0}^{+}}\;\text{and}\;t=\frac{{\hat{u}}_{N+1}^{+}}{{\hat{u}}_{0}^{+}} \end{array}$$

In optics, the flux balance related to a far field surface surrounding the whole multilayer leads to

5.6 $$\Phi_{0}^{+}=\Phi_{0}^{-}+\Phi_{S}^{+}+\Phi_{0}^{+}A,$$

with *Φ* the Poynting fluxes which are incident $\left(\Phi_{0}^{+}\right)$, reflected $\left(\Phi_{0}^{-}\right)$ and transmitted (*Φ*^+^_*S*_), and *A* the normalized absorption. For an elementary plane wave, these fluxes are proportional to $\Phi =\;\text{real}(m){\left|{\tilde{u}}^{\pm }\right|}^{2}$, so that the flux balance can be rewritten for the surface *Σ* as

$$1=R+T+A,\;\text{with }R={\left|r\right|}^{2}\;\text{and}\;T=\left[\frac{\text{real}(m_{s})}{\text{real(}m_{0}\text{)}}\right]{\left|t\right|}^{2}.$$

The heat balance is slightly different in the sense that, owing to heat diffusion, we cannot consider a source located at infinity (z=−∞), so that the far field position of the source is not valid and may underpin the separation between incident and reflected fluxes. However, the top interface temperature *T*_0_ is assumed to be forced (and known), so that the whole field distribution can be obtained within the stack, from the sequence (5.2). Then, the spectral quantity which we will analyse here is the ratio $t_{\mathrm{th}}={\hat{T}}_{S}^{+}/{\hat{T}}_{0}=t/(1+r),$ with ${\hat{T}}_{S}^{+}$ the field at the substrate interface. Hence, in the figures of this section, all fields are normalized to ${\hat{T}}_{0.}$

Now, some elementary or preliminary remarks are useful before numerical calculations. Indeed, numerous tools were developed in optics to design optical coatings and address specific challenges, which can be briefly addressed here in conjunction with heat conduction.

### (a) Preliminary remarks

#### (i) Single boundary

Consider first the case of a single boundary. As in optics, reflection is given by

5.7 $$r=\frac{m_{0}-m_{s}}{m_{0}+m_{s}}=\frac{-j\alpha_{0}b_{0}+j\alpha_{s}b_{s}}{-j\alpha_{0}b_{0}-j\alpha_{s}b_{s}}.$$

For a unique *z*-dependence (analogous to normal incidence), we obtain

5.8 $$\alpha_{i}=k_{i}=(1+j)\surd (\omega /2a_{i})\Rightarrow r=\frac{\beta_{0}-\beta_{1}}{\beta_{0}+\beta_{1}}$$

with *β*_*i*_ the effusivity

5.9 $$\beta_{i}=b_{i}/\surd a_{i}$$

This last equation shows that, when the calculation of reflection is considered, the effusivities in heat conduction play the role of the refractive indices in optics. This is valid at normal incidence, that is, for a zero spatial frequency (*σ*=0). It is interesting to note that the range of effusivities is much larger in conduction (more than two decades) than in optics (half a decade). Besides from that, temperature reflection from a single boundary is a real quantity. We also obtain 1+*r*=*t*⇒*t*_*th*_=1.

#### (ii) Specific multilayers

Absentee (half-wave) layers and quarter-wave stacks play a key role in multilayer optics [33–38], owing to their stationary properties. For these stacks, the phase term *δ* in the matrix (3.11) must be real and a multiple of *π*/2, which strongly simplifies the matrix and allows for an analytical design. However, this is only valid for quasi-transparent materials (*δ* is a real number), whereas the analogy between optics and conduction is for metallic layers. Therefore, the concept of quarter-wave stacks does not hold for conduction.

We could also wonder whether other concepts in metal optics could still be used for conduction. For instance, induced transmission filters [33–37] combine dielectric and metallic films to produce narrow-band filters with large rejection bands; with such devices, the optical field can be confined within the spacer (cavity) layer in a way similar to all-dielectric Fabry–Perot filters. Unfortunately, this will not be allowed for conduction multilayers: indeed, induced transmission filters require mixing metallic and dielectric layers, whereas for the optics/conduction analogy, all materials should be metallic with identical real and imaginary indices.

#### (iii) Field extrema

Another question concerns the ideas of field confinement and enhancement [47–49], which are major challenges in optics. Indeed, in optics, the harmonic field can spatially oscillate in the stack and specific coatings can be designed to reach huge enhancement of excitation in particular layers. In conduction, this would involve a local maximum of temperature within the stack, which is not possible in the absence of sources according to thermodynamics.

This result can be directly recovered from the governing equation (3.20) in the second Fourier plane, which can be turned into

$$b{\hat{u}}^{*}\frac{\partial^{2}\hat{u}}{\partial z^{2}}+b\alpha^{2}(\sigma ,\omega ){\left|\hat{u}\right|}^{2}=0,$$

which, after *z* integration, leads in each medium to

5.10 $${\left[b{\hat{u}}^{*}\frac{\partial \hat{u}}{\partial z}\right]}_{z1,z2}-\int_{z}{b\left|\frac{\partial \hat{u}}{\partial z}\right|}^{2}+\int_{z}b\alpha^{2}(\sigma ,\omega ){\left|\hat{u}\right|}^{2}=0,$$

where the first term within the brackets is continuous. Then, the presence of a maximum would allow one to choose these brackets between the *z*_1_ location of the extremum and *z*_2_ rejected at infinity where the field is zero. The result would be

5.11 $$\int_{z}b{\left|\frac{\partial \hat{u}}{\partial z}\right|}^{2}=\int_{z}b\alpha^{2}(\sigma ,w){\left|\hat{u}\right|}^{2}$$

which cannot be satisfied either for real or imaginary parts, because *α*^2^=*jω*/*a*−*σ*^2^.

Such a result (no temperature extrema in the absence of sources) does not contradict the fact that thin metallic layers create interferences in optics and allow the field to oscillate with depth location; indeed, one can check that optical oscillations vanish when the real and imaginary indices of the metal are identical, which underpins the analogy with conduction. For similar reasons, one can show that optical phenomena such as total internal reflection and total absorption [47–49] are not achieved in conduction.

### (b) Numerical results

For numerical calculation, we considered two materials with different thermal properties. This allows to keep the analogy with optical multilayers most of which involve two materials, that are denoted H (high refractive index *n*_H_) and L (low refractive index *n*_*l*_), with thicknesses of the order of a quarter-wavelength (*n*_H_*e*_H_=*λ*_0_/4,*n*_*l*_*e*_L_=*λ*_0_/4). Hence, the two thermal materials were chosen with different effusivities *β*_H_ (high) and *β*_L_ (low), that are

$$b_{H}=418\;\text{W}\;m^{-\text{1}}\;{\text{K}}^{-1}\;a_{H}=1.71\times 1{\text{0}}^{-\text{4}}m^{2}\;s^{-1}=>\beta_{H}=31965\;J\;{\text{K}}^{-1}\;m^{-\text{2}}\;s^{-0.5}\;\text{for}\;\text{silver,}$$

and

$$b_{L}=1.5\;\text{W}\;m^{-1}\;{\text{K}}^{-1}\;a_{L}=7\times 10^{-7}\;{\text{m}}^{2}\;s^{-1}=>\beta_{L}=1793\;J\;{\text{K}}^{-\text{1}}\;{\text{m}}^{-\text{2}}\;s^{-0.5}\;\text{for}\;\text{fused}\;\text{silica}.$$

Moreover, for each material, the thicknesses *e*_H_ and *e*_L_ were defined with respect to the thermal diffusion lengths at a design pulsation *ω*_0_=1 Hz, that are: *L*_H_=√(2*a*_H_/*ω*_0_)=1.85 cm and *L*_L_=√(2*a*_L_/*ω*_0_)=1.18 mm. The thickness values are a quarter of thermal length: *e*_H_=*L*_H_/4,*e*_*B*_=*L*_L_/4. Hence, the stack under study is a nine-layer coating of design: superstrate/*e*_H_*e*_L_*e*_H_*e*_L_*e*_H_*e*_L_*e*_H_*e*_L_*e*_H_/substrate, where both superstrate and substrate are quartz.

**Figure 2.**
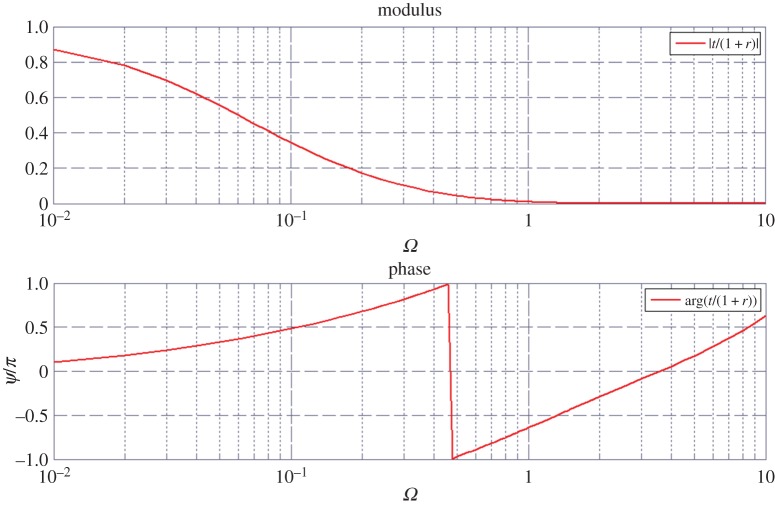
Spectral thermal transmittance versus pulsation, in modulus and phase. The horizontal units are in Hz. The multilayer is a nine-layer stack designed at pulsation *ω*_0_=1 Hz.

Then, using the optical admittance software, we investigated the stack thermal properties versus *z* location and temporal frequency (*ω*), at a given spatial frequency *σ*=0. Figure 2 is given for the spectral transmittance *t*_*th*_(*ω*) and emphasizes a classical behaviour versus increasing frequencies. The frequency range is [10^−2^,10 Hz].

**Figure 3.**
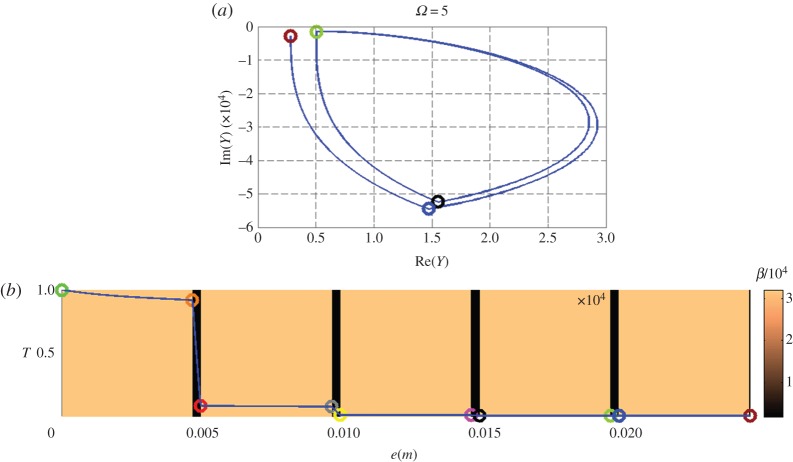
Nine-layer stack at pulsation *ω*=5 Hz, with the admittance diagram in the complex plane (a) and the distribution of temperature modulus (b). See text for all scales.

**Figure 4.**
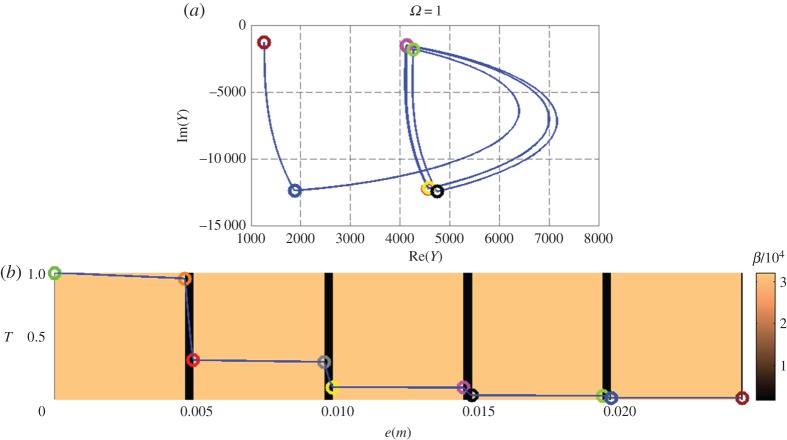
Nine-layer stack at pulsation *ω*=1 Hz, with the admittance diagram in the complex plane (a) and the distribution of temperature modulus (b). See text for all scales.

**Figure 5.**
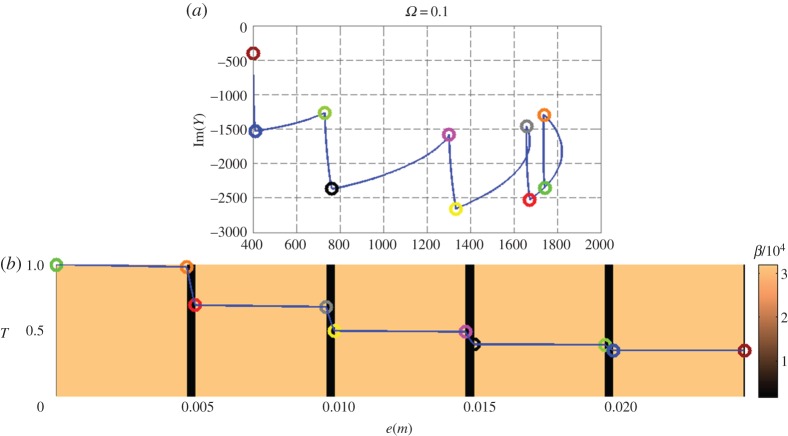
Nine-layer stack at pulsation *ω*=0.1 Hz, with the admittance diagram in the complex plane (a) and the distribution of temperature modulus (b). See text for all scales.

Figures 3–5 (top and bottom panels) are plotted at decreasing working frequencies, that are *ω*=5,*ω*=1 and *ω*=0.1 Hz. For each frequency, the bottom curve gives the normalized temperature distribution in modulus within the stack (left vertical scale), with a colour scale (right vertical scale) referring to the effusivity of each layer. The admittance diagram can be found in the top panels, with colour circles that enable to connect the admittance position in the complex plane to the altitude within the stack in the bottom panels.

The temperature modulus $\left|{\hat{u}}_{i}\right|$ is defined in the second Fourier plane from relation (3.21). Because a single temporal (*ω*) and spatial frequency (*ν*) are here involved, it gives in degree Kelvin the amplitude of the time variations of the one-dimensional harmonic temperature *u*_*i*_(*z*,*t*) as follows

$$\begin{array}{rl} & {\hat{u}}_{i}(\nu =0,z,\omega )=|{\hat{u}}_{i}|\;\text{exp}(j\varphi_{i})=>u_{i}(z,t)=2\text{ real}\{{\hat{u}}_{i}\text{exp[}-j\omega t\text{]}\}=2|{\hat{u}}_{i}(\nu =0,z,\omega )|\;\text{cos}[\omega t\\ & -\varphi_{i}(\nu =0,z,\omega )]\Rightarrow |{\hat{u}}_{i}(\nu =0,z,\omega )|=\left(\frac{1}{4}\right)[{\text{max}}_{t}u_{i}(z,t)-{\text{min}}_{t}u_{i}(z,t)]. \end{array}$$

From a practical way, the static temperature should be superimposed to *u*_*i*_(*z*,*t*). Furthermore, as mentioned in the introduction, the temperature ${\hat{u}}_{i}$ is normalized to its value at the top interface where it is forced, so that knowledge of the source is not required. Note that forcing the temperature can be reached with surface or bulk sources, as discussed in §6.

It was also necessary to check the energy balance of conduction given in (4.11). To do that, we chose a region free of sources, that is, the stack delimitated by the top interface (in contact with the superstrate) and the bottom interface (in contact with the substrate). Within this domain, relation (4.11) becomes

**Figure 6.**
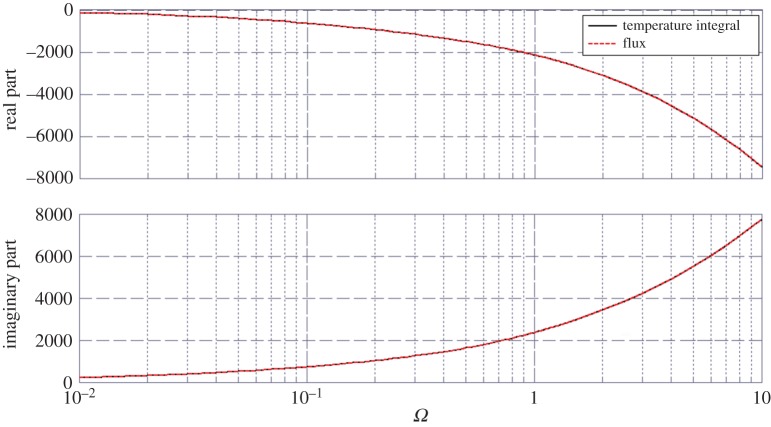
Validation of the complex conduction balance given in relation (5.12). Solid curve and dotted curve are superimposed.

5.12 $$\hat{S}=0\Rightarrow {\left[Y\hat{T}\right]}_{z}=\int_{z}b\alpha^{2}\hat{T}\text{d}z,$$

which means that because no heat power is provided within the domain, the bulk temperature elevation is responsible for the surface flux variation between the top and the bottom surfaces of the stack. In figure 6, both real and imaginary parts of these two terms (flux and temperature integral) are plotted and the results show a perfect agreement. We also checked that the agreement holds regardless of the spatial frequency *σ*. Note again that the temperature was normalized to its value at the top interface, and that the flux modulus is given in W m^−2^.

### (c) From metallic to transparent optical multilayers

**Figure 7.**
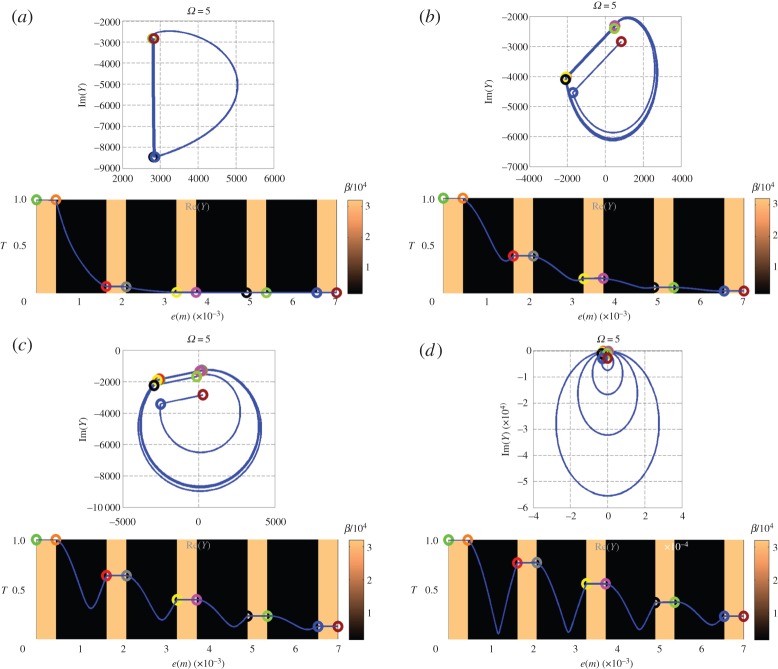
(*a*−*d*) Nine-layer metallic stack at pulsation *Ω*=5 Hz; Modification of admittance diagram and electric field distribution when the imaginary part of the *k* parameter is decreased from *k*^′′^=*k*^′^ to *k*^′′^=0 (see text for more details). The specific admittance circles of dielectric optics are recovered in the limit of vanishing *k*^′′^.

To complete this section, figure 7*a*−*d* illustrates how the admittance diagrams and the field distribution of metal optics (which we used for conduction) approach those of dielectric multilayers (where the analogy with conduction breaks down) when the imaginary index is decreased. To achieve this correspondence between admittance diagrams in heat conduction and light propagation, we considered a thermal multilayer with conduction number *k*=*k*^′^+*jk*^′′^, and plotted the admittance diagrams at *Ω*=5 Hz for decreasing values of *k*^′′^. These values are *k*^′′^=*k*^′^ (top left panel), *k*^′′^=0.3*k*^′^ (top right panel), *k*^′′^=0.1*k*^′^ (bottom left panel) and *k*^′′^=0 (bottom right panel). The thicknesses are *e*_H_=*L*_H_/40 and *e*_L_=*L*_L_ at design frequency *Ω*_0_=1 Hz. These panels confirm that specific admittance circles are progressively recovered when the media become transparent, which is a well-known fact in thin film optics [33]. Also, the field oscillations within the stack are recovered when the imaginary index vanishes, in agreement with (5.11).

## 6. Microcavities

Optical formalisms developed for luminescent microcavities also remain valid for heat conduction. Until now, multilayers were excited with a source in free space in the superstrate. By opposition with this far field geometry, the same devices are known as microcavities when they support the sources in their bulks. The optical (linear) solution [48,49] remains strongly similar whatever the position of the source (far or near-field), provided that the field discontinuities are corrected, that is, **z**^∧^*δ***E**=**z**^∧^*δ***H**=**0** for optical coatings (far field), and **z**^∧^*δ***E**=**0**,**z**^∧^*δ***H**=**J** for optical microcavities (near-field) with **J** a surface electric current. Extension of the admittance formalism [48,49] then gives the solution in the second Fourier plane as

6.1 $${\hat{u}}_{i}={\hat{u}}_{i}^{\prime }=\frac{\hat{J_{i}}}{Y_{i}^{\prime }-Y_{i}}$$

where ${\hat{u}}_{i}$ and ${\hat{u}}_{i}^{\prime }$ are the fields on each side of interface *i* in media *i*−1 and *i*, respectively, and $\hat{J_{i}}$ the electric current density at surface *i* which creates these fields. The admittances characterize each half part of the stack; they are still calculated from (5.1), but their initial values of effective index are taken in the substrate for Y^′^ and in the superstrate for *Y* [48,49]. The field sequence (5.2) can then be used to obtain the field values ${\hat{u}}_{0}^{\pm }$ in the extreme media. After summation over all currents, the result is

6.2 $${\hat{u}}_{0}^{-}=\sum_{i}\frac{C_{i}^{-}{\hat{J}}_{i}}{Y_{i}^{\prime }-Y_{i}}\;\text{and}\;{\hat{u}}_{0}^{+}=\sum_{i}\frac{C_{i}^{+}{\hat{J}}_{i}}{Y_{i}^{\prime }-Y_{i}},$$

where $C_{i}^{\pm }$ are optical factors which are design dependent, and that allow to reach the superstrate $\left(C_{i}^{-}\right)$ or the substrate $\left(C_{i}^{+}\right)$.

Consider now the case of thermal microcavities, in the form of planar multilayers supporting thermal sources at theirs interfaces. The conduction process of such devices will again behave like optical cavities in metals, because the discontinuities for temperature and flux are again given in a way similar to optics, that is, for a surface source *S*_*i*_ at interface *i*

6.3 $$\delta T_{i}=0\;\text{and}\;\delta h_{i}=S_{i}\Rightarrow T_{i}^{\prime }=T_{i}=\frac{\hat{S_{i}}}{Y_{i}^{\prime }-Y_{i}}.$$

This last relationship is identical to (6.1) and allows one to address thermal microcavities with the same optical softwares, provided that the scalar thermal sources (*S*_*i*_) replace the algebraic electric currents *J*_*i*_. Contrary to the far field geometry, the thermal field now can oscillate within the stack, owing to the presence of thermal sources. In the case of bulk sources, the field would follow the source at high frequencies. Note, however, that optics involves the intensity (the field square), which introduces additional interaction terms $\left({\hat{J}}_{i}{\hat{J}}_{j}^{*}\right)$ between the currents [48,49]. To conclude this section, we note that the power (optical or thermal) provided by the confined source within the stack depends on the multilayer geometry [48,49].

## 7. Diffraction gratings

Optical diffraction by a periodic surface is well known to spread the incident energy into a discrete series of spatial frequencies given by

7.1 $$\sigma_{m}=\sigma_{0}+\frac{q}{d},$$

with *d* the grating period, *σ*_0_ the incident spatial pulsation and *q* a relative integer. In terms of scattering angles *θ*_*p*_ in air, the result is

7.2 $$\text{sin}\theta_{q}-\text{sin}i_{0}=q\frac{\lambda }{d},$$

with *λ* the wavelength and *i*_0_ the illumination angle. Several techniques exist to predict the angular efficiency at each diffraction order m, like modal and differential methods, finite-elements and boundary integrals.

The same computer codes can again be applied for diffraction of heat conduction, a process that makes temperature only significant at specific frequencies or directions given by (7.1) and (7.2). However, unlike for optics, one must take account of the attenuation resulting from thermal diffusion, which confines heat diffraction in the vicinity of the grating. Moreover, because diffraction does not occur for subwavelength gratings, the grating period at normal illumination (excitation) would be greater than one wavelength (*d*>*λ*); such a condition is seemingly trivial in optics but not in conduction, where wavelength and thermal length are similar (*λ*=2*πL*). Indeed, the consequence is that the conduction diffraction process will be significant at a distance *z* of the average surface lower than its period, that is, *z*<*L*<*d*/2*π*. Finally, depending on the thermal length value *L*, far field effects can be masked or not, which forces diffraction to be emphasized in the near-field.

**Figure 8.**
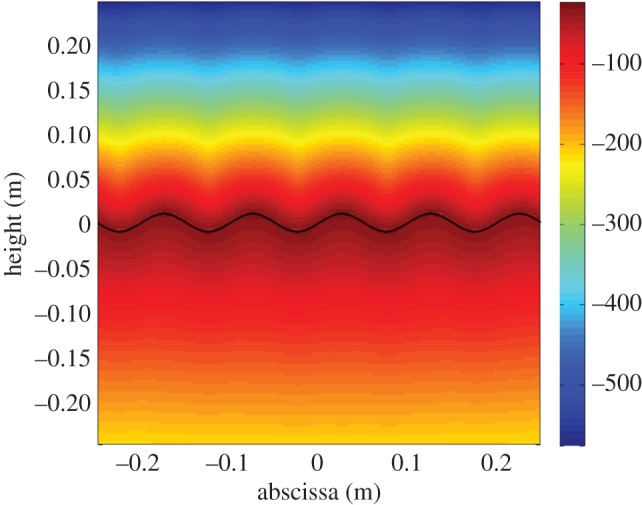
Diffraction of a conduction process. The full line is for the sinusoidal surface which separates air (top medium) from silver (bottom medium). The vertical units on the right are for the grey levels and related to the temperature modulus in dB.

**Figure 9.**
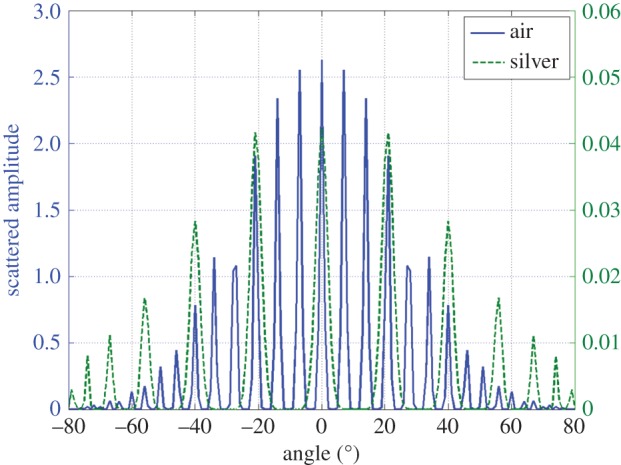
Spatial harmonics of the temperature modulus (grating law).

Numerical calculation is given in figures 8 and 9, at a thermal pulsation of 10 Hz. The grating is a surface with a sinusoidal shape graved at the top of a silver (Ag) substrate, with an amplitude of 1 cm and a period of 10 cm. The superstrate is air. The heat source results from the absorption of an optical beam of 37.5 cm in size, at normal incidence and 633 nm wavelength. The complex refractive index of Ag at this wavelength is chosen as *n*_*Ag*_=0.134+3.99*i*. Owing to the low depth penetration of optics within the metal, such heat source can be assumed to be located at the grating surface. Moreover, the thermal parameters of Ag are 1.71×10^−4^ m ^2^ s^−1^ (diffusivity) and 418W (mK)^−1^ (conductivity).

The boundary integral formalism for the electromagnetic wave scattering from one-dimensional rough surfaces [51,50] has been adapted to this heat conduction problem. The temperature, which is set continuous across the grating surface, and the normal component of the heat flux, which discontinuity identifies with the heat source, are the unknown functions of a set of coupled boundary integral equations. These equations are numerically solved with the method of moments [51,50] using piecewise-constant basis functions and point-matching testing functions. The discretized grating surface is 3 m long, with a sampling step of 1.5 mm.

Figure 8 gives the two-dimensional modulus of the temperature distribution in the substrate and superstrate. As expected, the field shows periodic variations in both media near the centre of the beam footprint. However, the harmonics which are specific of the grating law cannot be emphasized in figure 8, due to the fact that calculation is for the near-field, which requires to superimpose all harmonics

7.3 $$\overset{ˇ}{u}(x,z,\omega )=\sum_{q}{\overset{ˇ}{u}}_{q}(x,z,\omega ).$$

To go further, we have plotted in figure 9 the modulus of the different grating harmonics ${\overset{ˇ}{u}}_{q}$ in the superstrate and in the substrate. The results emphasize the validity of the grating law (7.2) in conduction.

**Figure 10.**
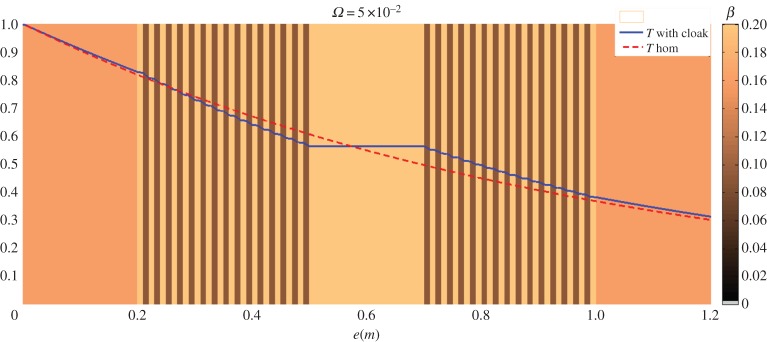
Modulus of temperature distribution at 0.05 Hz inside and outside the multilayer planar cloak surrounding a spacer layer. The field for the homogeneous medium is plotted as a dashed line and the field for the highly conducting obstacle surrounded by specially designed stacks (the cloak) is plotted as a full line.

In figures 9 and 10, the incident optical power is chosen to 1 W. Because the silver absorption is around 3%, the absolute absorption responsible for the heat source is around 30 mW. From a practical point of view, the harmonic temperature could be increased by a factor 30 when using an absorbing black coating (99% absorption), provided no damage threshold occurs owing to the total temperature. Note that beam focusing would not help, because several periods of the grating must be illuminated. Furthermore, near-field probing would make easier the detection of temperature variations in the grating vicinity, where the thermal attenuation is weak. The grating period can also be increased to allow classical measurements.

Other diffraction processes can be performed in a similar way, including scattering from inhomogeneities (roughness or bulk) and Mie theory for spherical particles. The diffraction problem for heat conduction could also be addressed within the formalism of microcavities. Indeed, a surface thermal source can be created by the deposition of a very thin (less than *λ*/50) metallic layer on a dielectric material, followed by an etching process with a period *d*. Under optical illumination, optical absorption in this device will only be concerned with the metallic patterns. When the beam is modulated at frequency *ω*, the resulting heat source is

7.4 $$S(x,\omega )\approx A(\omega )h(x)*\sum_{q}\delta (x-qd)=A(\omega )\sum_{q}h(x-qd),$$

with *δ* the Dirac distribution, *h* the shape of isolated pattern and *A* an absorption term. Then, Fourier transformation allows one to retrieve the grating law in the form

7.5 $$\hat{S}(\nu ,\omega )=A(\omega )\sum_{q}\hat{h}\left(\frac{q}{d}\right)\delta \frac{\nu -q}{d},$$

and heat diffraction can be directly calculated following (6.3).

## 8. Towards a multilayer planar cloak

The last example in the analogy concerns transformation optics, a tool currently used for cloaking in different fields including heat [16–32,52,53]. Invisibility cloaks usually have circular or spherical geometries, while we keep here the planar geometry of previous sections and consider a single layer that should be ‘hidden’ with the help of additional symmetrical stacks on its left and right sides. In order to design such a planar cloak, we apply a geometric transform in the spirit of Kohn *et al.*[53] which maps the disjoint interval [−*e*_1_,−*η*] and [*η*,*e*_1_] (where *η*<*e*_1_ is a small positive parameter) onto [−*e*_1_,−*e*_2_] and [*e*_2_,*e*_1_] with *e*_2_<*e*_1_. Similarly, the small interval (−*η*,*η*) is mapped onto (−*e*_2_,*e*_2_). When *η* tends to 0, this transform amounts to mapping [−*e*_1_,*e*_1_]∖{0} onto intervals [−*e*_1_,−*e*_2_) and (*e*_2_,*e*_1_] in the spirit of Pendry *et al.* [29].

While the two transforms make sense in two-dimensional and three-dimensional cases, the former one (Kohn's transform) seems to be more natural in our one-dimensional configuration. With a linear transformation (*x*^′^=*αx*+*β*,*y*^′^=*y*), we obtain an ideal anisotropic planar cloak surrounding a central (spacer) anisotropic layer. All media (cloak and spacer layer) are anisotropic following the transformation. The conductivity matrix (*b*_*ij*_) of the cloak is diagonal with *b*_11_=(*e*_1_−*e*_2_)/(*e*_1_−*η*) and *b*_22_=(*e*_1_−*η*)/(*e*_1_−*e*_2_), whereas that (*b*′_*ij*_) of the spacer layer is also diagonal with *b*′_11_=*e*_2_/*η* and *b*′_22_=*η*/*e*_2_. In order to achieve these required anisotropic media, we consider an alternation of layers with isotropic conductivities which behave like an effective medium with anisotropic conductivity, see references [16,18] for construction of circular counterparts of the planar cloak with homogenization techniques. The result for the cloak is an alternation of isotropic layers of two materials with equal thicknesses and conductivities given at *η*=0 by

8.1 $$b_{1}=\left[\frac{1}{e_{1}-e_{2}}\right]\{e_{1}+{\left[e_{2}(2e_{1}-e_{2})\right]}^{0.5}\}\;\text{and}\;\;b_{2}=\left[\frac{1}{e_{1}-e_{2}}\right]e_{1}-{\left[e_{2}(2e_{1}-e_{2})\right]}^{0.5}$$

**Figure 11.**
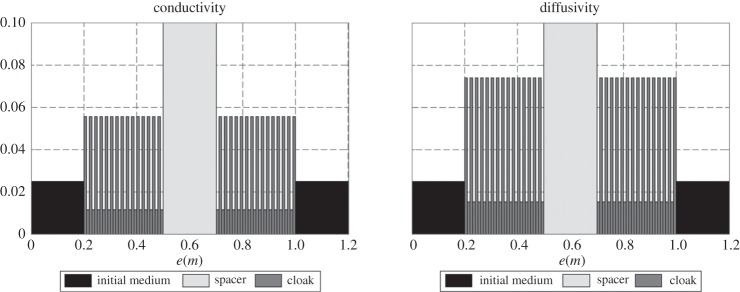
Stack parameters of figure 10 (conductivities on the left, diffusivities on the right). The thermal parameters are out of scale for the spacer layer (see text).

However, although this homogenization process works for the cloak, this is not the case for the spacer layer. Because the spacer conductivity matrix involves two real diagonal terms that are real infinite and zero, respectively, the transparency effect with an isotropic spacer layer can only be reached for very large conductivities inside the spacer layer. This prediction is confirmed in figure 10, because the temperature modulus (similar to that of figures 3–5) outside the cloak is nearly superimposed to that of the initial homogeneous medium. In figure 10, the spacer layer conductivity is far greater (several decades) than the other conductivities (i.e. 10^5^ higher than that of the initial medium). Complete geometry of the multilayer stack is given in figure 11.

These results confirm that transformation optics still remain valid in the case of heat diffusion. Clearly, here, cloaking is limited to some very specific limiting case, owing to the high conductivity required within the spacer layer, but the mathematical technique remains the same.

## 9. Static versus harmonic regimes

The matrix formalism (3.10) developed above works whether the regime is static (*Ω*=0) or dynamic (*Ω*≠0). In the static regime, the matrix coefficients take a single form, and the resulting product matrix for the multilayer becomes

9.1 $$M=\left(\begin{array}{cc} 1 & e_{t}/b_{\mathrm{eq}}\\ 0 & 1 \end{array}\right)$$

with *e*_*t*_ the total thickness and *b*_*eq*_ the equivalent conductivity

9.2 $$\frac{e_{t}}{b_{\mathrm{eq}}}=\frac{\sum_{k}e_{k}}{b_{k}},$$

which is a well-known result. However, there is a key difference with the harmonic regime which concerns the admittances. Indeed, at non-zero frequencies, the admittances only depend on the multilayer design (geometry, optical or thermal parameters), whereas in the static regime, they also depend on the extreme fields in the form

9.3 $$Y_{s}=\left(\frac{b_{\mathrm{eq}}}{e_{t}}\right)\left(\frac{T_{0}-T_{s}}{T_{s}}\right)\;\text{and}\;Y_{0}=\left(\frac{b_{\mathrm{eq}}}{e_{t}}\right)\left(\frac{T_{0}-T_{s}}{T_{0}}\right).$$

This point recalls why the frontier temperatures (or fluxes) must be forced in the static regime, which is not required in the harmonic regime. This is related to the fact that the one-dimensional static solution in the extreme media free of sources involves two parameters (*T*=*αz*+*β*), whereas the harmonic solution requires one parameter (*T*=*T*_s_ exp (*jαz*)); in other words, with the harmonic regime the derivative of *T* is proportional to *T*, which is not the case for the static solution.

The analogy with optics still holds in the static regime, because electrostatics also involves a potential difference (rather than a potential), which is classical. However, in the harmonic regime (electromagnetism), there is no supplementary condition at the domain frontiers, so that the multilayer problem is entirely solved by the sequence relationship (5.1) of the admittances, which allows one to retrieve the top admittance *Y*_0_(*m*_s_) from the substrate one. However, in some situations like modal optics (guided waves), another condition must be fulfilled to force the field to only merge both in the superstrate and substrate, that is *Y*_0_(*m*_s_)=−*m*_0_. The result is a discretization of (5.1) which gives the guided modes as the poles of the reflection factor. A similar discretization would be obtained in the harmonic regime when temperature (or flux) is forced in both extreme media.

## 10. Conclusion

We have explored an analogy between optical propagation and heat conduction in isotropic, homogeneous and linear media. Both fields (scalar electric field and temperature) were shown to follow the same harmonic equation in the second Fourier plane, provided that the optical wavenumber $k_{\mathrm{opt}}=\omega \surd [\overset{ˇ}{\varepsilon }(\omega )\overset{ˇ}{\mu }(\omega )]$ is replaced by the heat conduction number *k*_th_=(1+*j*) √[*ω*/2*a*]. The dispersion law of this wavenumber has the memory of the time derivation order of the governing spatio-temporal equation. Moreover, the analytic expression of the fields, conduction and wavenumbers show that thermal diffusion is strongly analogous to optical propagation in metallic media; however, the optical refractive index of these artificial metallic media should have identical real and imaginary parts, a specific property where applications of metal optics can be reduced.

The case of planar multilayers was addressed, and the optical admittance formalism was shown to be directly applicable to thermal conduction. Within this framework, the temperature and algebraic heat flux played the role of electric and magnetic field tangential components, respectively. The only notable difference in the admittance formalism is the required modification of the optical effective index in order to take into account the heat conduction number (*m*_th_=−*jαb* replaces *m*_opt_=*nα*/*k*). Numerical calculations were presented to validate all results, including the optical and thermal energy balances.

The analogy between metal optics and conduction was then extended to microcavities and diffraction gratings, and to transformation optics. All results show that most softwares developed for metal optics can be directly used for conduction, whether they address interference filters or microcavities, diffraction gratings or photonic crystals, metamaterials or transformation optics. We finally note that Green's functions for reflected and thermal fields at planar interfaces were computed in reference [52] and they are reminiscent of what researchers compute in optics. However, one must keep in mind that these analogies only hold for specific metals (real index= imaginary index). We hope our work will foster theoretical and experimental efforts in thermal metamaterials.

## Data accessibility

All data and calculation methods are given in the manuscript, so that all results are fully reproducible.
